# The Leicester Guardians

**Published:** 1899-12-23

**Authors:** 


### The Hospital

A Journal of

The Medical Sciences and Hospital Administration.

Vol. XXVII.—No. 691.] Edited by Sir Henry Burdett, K.C.B., and Solomon C. Smith, M.D., M.R.C.P. [December 23, 1899.

### The Leicester Guardians.

When the illustrious members of the Pickwick Club fell into the hands of the law, as personified by Mr. Grummer and his myrmidons, and when the procession escorting them was interrupted by the appearance of Mr. Weller, it will be remembered that Mr. Snodgrass partly took off his coat, and announced in a loud voice that he "was going to begin." A similar line of conduct was adopted a few months ago by the Leicester Board of Guardians, who, in the course of last June, were summoned before the Queen's Bench Division of the High Court, at the instance of the Local Government Board, to answer for the offence of having failed to comply with the Act of Parliament which directed them to appoint a vaccination officer. The case was heard in July, and a mandamus issued, directing the defendants to make the appointment. Of their deportment in Court on the occasion we have no record, and it may not improbably have been what is commonly known as sheepish; but the public spirit and intelligence of Leicester, as represented by a rabble said to have been ten thousand in number, turned out to welcome them on their return, and escorted them to a building described as the "Floral Hall," where as many as possible of the sympathisers constituted themselves into a public meeting, of which Sir Israel Hart was chairman, and proceeded, after a vocal performance of what were described as "Anti-Vaccination Hymns" (we should like to see that hymnal), to pass a series of absurd and bombastic resolutions. The defendants were forty in number, and five of them were ladies, who naturally took a leading part in the Floral Hall performance. One of these ladies announced her own determination, and that of her colleagues, to "defend the liberties of the country," and the whole of the party declared their preparedness—not to say their eagerness—to undergo imprisonment for "a considerable period" rather than yield obedience to the law, which, like Mr. Bumble, they considered to be acting indefensibly in requiring them to pursue a course which was in direct opposition to what they facetiously described as their "convictions." It apparently had not occurred to them that the course best calculated to satisfy these convictions would have been to decline acceptance of an office which involved the performance of duties opposed to them. Two days after the public meeting there was an ordinary meeting of the Board; and the business of appointing a Vaccination Officer had a place upon the list of agenda. Even in that short period, the hearts of two of the male recalcitrants had descended into their boots; and they basely voted in favour of submission. We have not heard what punishment, if any, they received at the hands of the colleagues whom they had abandoned, or of the fellow townsmen whose confidence they had betrayed; but it was at any rate insufficient to check contagion from their example, and the prospect of the consequences of contempt of court became less and less inviting as they approached.

A time actually arrived at which seventeen of the Guardians were ready to make the required appointment, leaving only twenty-three to undergo the pains and to reap the glories of martyrdom. And the worst of the position was that while the twenty-three were in prison, purging themselves of their contempt, the seventeen would appoint the officer, and the original incident would be closed. So, like an Irishman famous in story, they bethought themselves of a "skame." They would appoint the most prominent anti-vaccinator they could find, and would trust to him to leave his duties unperformed. Their choice fell upon a gentleman named Stephen, of whom we only know that he has been described as a local journalist, and that he was thought likely to act as the Guardians desired. But the cruel Local Government Board again intervened, and the Guardians were once more haled before the Queen's Bench Division, where Justices Darling and Channell declined to be "trifled with by an illusory appointment," and ordered a writ of attachment against the defendants to be prepared, and to issue on the fifth day of next term, that is, on the fifteenth of January, unless in the meantime a proper officer, satisfactory to the Local Government Board, had been appointed. Then came the collapse. The "liberties of the country" were suffered to perish. The rhetoric of the Floral Hall was forgotten, or was proved to be as empty as the *crania* of those anti-vaccinators who do not make a living out of the business. The counsel for the defendants apologised to the Court in the humblest possible manner, and undertook, on the part of his clients, that the requirements of the mandamus should be strictly complied with, in the spirit as well as in the letter, within the limit of time indulgently afforded for the purpose. The curtain, we may presume, has now fallen upon the last act of the sorry farce, leaving only the lesson that it would be very desirable to transfer the entire control of vaccination from the Poor Law to the Medical Department of the Local Government Board, and to make sanitary authorities primarily responsible for the appointment of the superintending officer. The whole business appertains to the preservation of the public health, rather than to the relief of the poor.

